# Nanoscale pore structure and fractal characteristics of lacustrine shale: A case study of the Upper Cretaceous Qingshankou shales, Southern Songliao Basin, China

**DOI:** 10.1371/journal.pone.0309346

**Published:** 2024-10-18

**Authors:** Changjun Ji, Tianfu Liu, Yun Chen, Qian Wang, Peng Sun, Lei Sun, Taohua He

**Affiliations:** 1 Chinese Academy of Geological Sciences, Beijing, China; 2 PetroChina uSmile Company Limited, Beijing, China; 3 School of Petroleum Engineering Yangtze University, Wuhan, China; 4 Hubei Key Laboratory of Petroleum Geochemistry and Environment (Yangtze University), Yangtze University, Wuhan, China; 5 Shenyang Geological Survey Center, China Geological Survey, Shenyang, China; Soran University, IRAQ

## Abstract

The Upper Cretaceous Qingshankou Formation’s lacustrine shales in the Songliao Basin are among China’s most promising shale oil reservoirs. To elucidate their pore and fractal characteristics, a comprehensive set of analyses encompassing total organic carbon (TOC), X-ray diffraction (XRD), and low-temperature N_2_ adsorption (LTNA), Rock-Eval pyrolysis experiments and two-dimensional nuclear magnetic resonance (2D-NMR) were conducted. Using the Frenkel-Halsey-Hill (FHH) method, fractal dimensions (D) were calculated, and their relationship with pore metrics and shale compositions were explored. Two distinct fractal dimensions, D_1_ (0 < P/P_0_ < 0.5) and D_2_ (0.5 <P/P_0_ <1.0), were derived from LTNA isotherms via the FHH approach. D_1_ values fluctuated between 2.5715 and 2.7551 (mean 2.6564), while D_2_ spanned from 2.3247 to 2.4209 (mean 2.3653). Notably, D_1_ consistently surpassed D_2_, signifying that smaller pores exhibit greater homogeneity compared to their larger counterparts. D_1_ gradually increases with the increase of clay content. A direct correlation was observed between pore volume (PV), specific surface area (SSA), and D (both D_1_ and D_2_), whereas the association between average pore diameter (APD) and D was inverse. Both D_1_ and D_2_ escalated with diminishing TOC, 2D-NMR solid organic matter (OM), S_1_ content and 2D-NMR light oil. Intriguingly, D_1_ showed a stronger association with key pore and "sweet spot" parameters, highlighting its utility in assessing pore structural complexity and shale oil potential. This study illustrates how fractal theory enhances our understanding of pore structures and the shale oil enrichment process for the lacustrine shale.

## 1. Introduction

The escalating global energy demand has catalyzed a renewed interest in unconventional oil and gas exploration, and predecessors enhanced the lacustrine shale study by incorporating perspectives from remote sensing and artificial intelligence [1–6]. Notably, the pioneering advancements in the exploration and extraction of lacustrine shale within the Songliao Basin have positioned shale oil at the forefront of energy discussions, in response to the burgeoning global energy requirements [7–12]. The intricate pore architecture of shale oil reservoirs, characterized by pronounced heterogeneity, diverse pore classifications (interparticle, intraparticle, organic matter, and microfracture), and an extensive pore size distribution (PSD), plays a pivotal role in dictating the storage and migratory capacity of shale oil [11, 13–15].

Shale reservoir pore configurations encompass aspects such as pore typology, dimensions, morphology, PSD (pore size distribution), PV (pore volume), SSA (specific surface area), and pore interconnectivity [16–18]. Techniques to delineate shale pore topography predominantly fall into three categories: imaging techniques, fluid injection methodologies, and X-ray procedures. Imaging techniques comprise advanced scanning electron microscopy variants (e.g., FE-SEM, BIB-SEM, FIB-SEM) and nano-micro CT, renowned for their high-resolution capabilities and direct pore morphological visualization [19, 20]. Fluid injection techniques, encompassing low-temperature gas adsorption and pressure-regulated porosimetry, excel in characterizing interconnected pore structures [14, 15, 21]. X-ray methodologies, including small-angle neutron scattering (SANS), ultra-small angle neutron scattering (USANS), low-field nuclear magnetic resonance (LF-NMR), and two-dimensional nuclear magnetic resonance (2D-NMR), offer distinct advantages [22–25]. For instance, SANS and USANS can ascertain pore structural parameters under variable temperature and pressure conditions, unhindered by occlusion effects or pore connectivity disturbances. LF-NMR offers rapid, non-destructive sample analysis, furnishing parameters like porosity, permeability, and PSD. The high-frequency 2D-NMR (T1-T2) technique can discern content information of diverse components. Recent scholarly endeavors have employed a multifaceted approach to delineate the pore structure of Qingshankou shales in the Songliao Basin. This investigation delves into the nano-micro heterogeneity attributes of Qingshankou shales, an area that warrants further exploration.

In this study, sixteen Qingshankou lacustrine shale samples from the southern Songliao Basin were meticulously analyzed using LTNA experiments and the FHH methodology. A holistic approach, integrating total organic carbon (TOC), X-ray diffraction (XRD), and low-temperature N_2_ adsorption (LTNA), Rock-Eval pyrolysis experiments and two-dimensional nuclear magnetic resonance (2D-NMR), was employed to discern correlations between fractal dimensions (D), pore structural metrics, and shale reservoir compositions. The findings offer invaluable insights into the labyrinthine pore networks in lacustrine shale reservoirs, serving as a scientific compass for the efficacious exploitation of shale oil.

## 2 Geological setting

**Fig 1:**
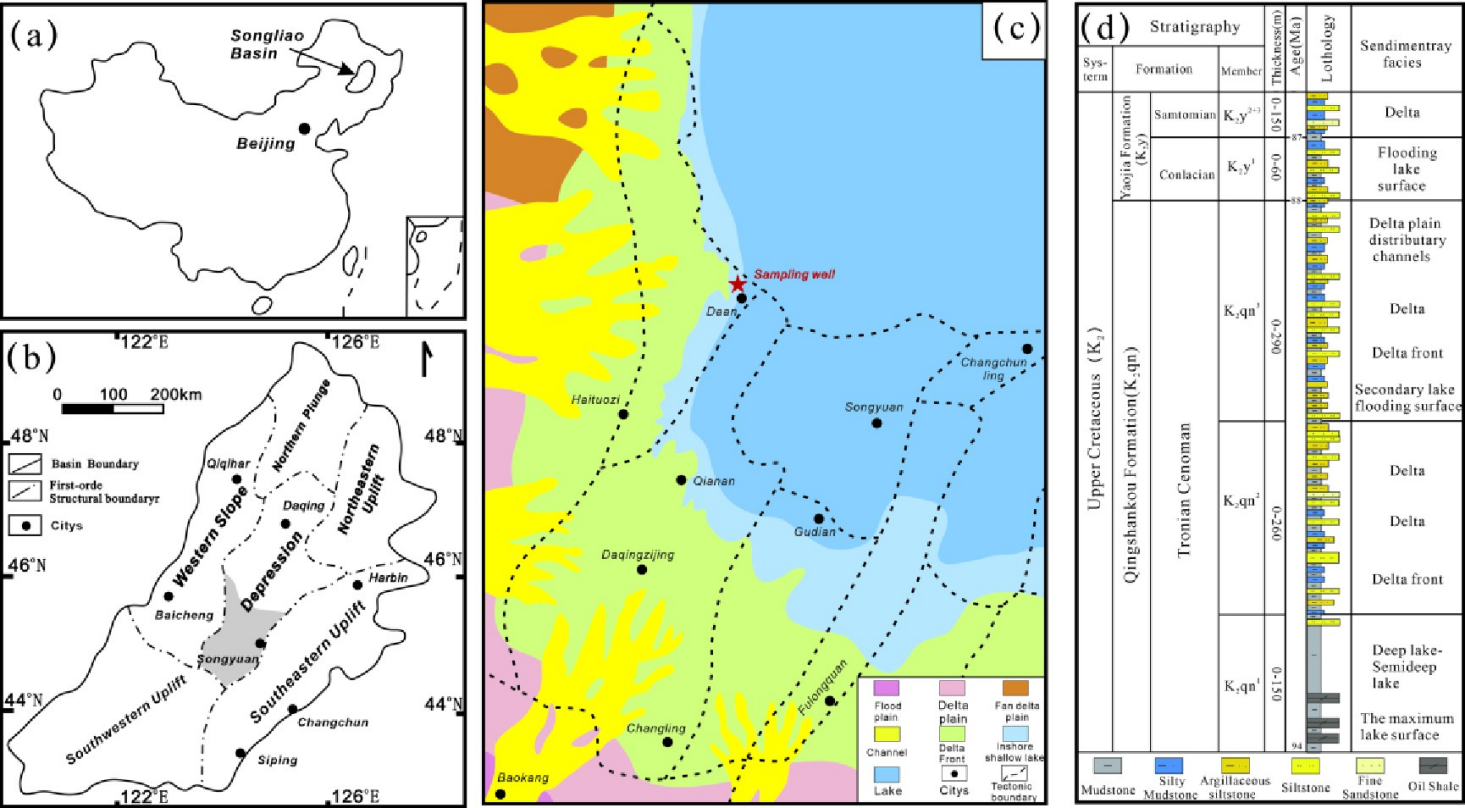
Location and the lithology column of the investigated well in Southern Songliao Basin, China (adapted from Ref. **[26]).** During the sedimentary epoch of the Qingshankou Formation, extensive aquatic incursions led to the deposition of copious layers of organic-rich dark mudstone and shale. The Qingshankou Formation not only serves as a source rock for conventional hydrocarbons but also emerges as a target for shale oil exploration endeavors [9, 27].

The vast Songliao Basin bifurcates into northern and southern territories, demarcated by the Songhua River [8, 26]. Owing to its unique geological characteristics, the Songliao Basin is segmented into six principal structural domains: the Western Slope, the Central Depression, the Northern Plunge, the Southeastern, Northeastern and Southwestern Uplifts (Fig 1) [26]. At the core of the basin lies the Central Depression, recognized as the principal locus for hydrocarbon generation and accumulation in the southern expanse of the basin, and identified as a promising area for shale oil exploration. This zone further subdivides into four tertiary structural entities: Fuxin Uplift, Changling Sag, Honggang Terrace, and Huazijing Terrace. The Changling Sag, situated in the southern part of the Central Depression, covers an area of approximately 6500 km^2^ and is bordered by the Gulong Depression to the north, with the Honggang and Huazijing Terraces to its northwest and southeast, respectively.

## 3 Materials and methods

### 3.1 Materials

In the southern region of the Songliao Basin, two distinct types of shale oil reservoirs are identified: the laminated shale oil reservoir and the massive shale oil reservoir. The laminated shale oil reservoir, positioned along the periphery of the basin, features a relatively shallow burial depth, interlayers of sandstone, and a considerable concentration of brittle minerals. This reservoir type is notably amenable to large-scale hydraulic fracturing operations, experiencing significant developmental progress in recent years. Conversely, the massive shale oil reservoir, situated at the core of the basin, is characterized by its deep burial depth and a substantial content of clay minerals, with developmental breakthroughs remaining elusive. Consequently, the exploratory well D86, centrally located within the basin, was selected for this investigation.

Fig 1 delineates the strategic placement of well D86 in the Changling Sag, situated in the southern sector of the Songliao Basin. This pivotal exploratory well, established in 2020, yielded sixteen lacustrine shale core samples from the primary stratum of the Qingshankou Formation (K_2_qn^1^), extracted at depths ranging between 1971.2 and 2007.0 meters. Comprehensive evaluations were conducted, including Rock-Eval pyrolysis, total organic carbon (TOC) determination, X-ray diffraction (XRD) analysis, and Low-temperature N_2_ adsorption (LTNA) assays. All the above experiments were completed at Hubei Key Laboratory of Petroleum Geochemistry and Environment, Yangtze University, Wuhan, China.

### 3.2. Geochemical evaluations

The complete collection of sixteen shale samples was meticulously cleaned and then finely ground to a granularity of 80 mesh using an agate mortar and pestle, setting the stage for subsequent pyrolysis and TOC analysis. After the elimination of carbonate and dolomite with 10% HCl at 85°C for 4 h for crushed samples (200 mesh size) in a crucible, the determination of TOC concentrations was conducted using a CS-230 carbon-sulfur analyzer, in strict compliance with the Chinese national standard GB/T 19145–2003. Pyrolysis analyses were carried out with a Rock-Eval 6 plus analyzer, adhering to the Chinese national standard GB/T 18602–2012. Key parameters, including S_1_, S_2_ and T_max_, were extracted from the pyrolysis data. Hydrogen Index (HI) was calculated by HI = S_2_/TOC*100.

### 3.3 X-ray diffraction (XRD)

For the discernment of mineralogical compositions, a D8 DISCOVER X-ray diffractometer was employed, leveraging Cu Kα-radiation (λ = 0.15418 nm). Operating parameters included a working voltage of 30 kV-45 kV, an operating current range of 20 mA-100 mA, and a 1 mm slit with a scanning velocity of 4/min. These procedures adhered to the Petro-China Standard SY/T 5163–2018.

### 3.4 Low-temperature N_2_ adsorption experiment (LTNA)

The LTNA analysis was conducted utilizing an ASAP 2460 surface area analyzer in adherence to the China National Standard GB.T 19587–2004. Prior to the LTNA assessment, all shale samples were milled to a granularity of 60–80 mesh and subsequently subjected to degassing in a vacuum oven at 110°C for approximately 16 hours to eradicate adsorbed contaminants and gaseous entities. Nitrogen adsorption/desorption isotherms were recorded at a temperature of 77.3K (-196°C), with the relative pressures (P/P_0_) oscillating between 0.01 and 1.

### 3.5 Two-dimensional nuclear magnetic resonance (2D-NMR) spectroscopy

An advanced NMR apparatus, designated as MesoMR23-060H-I and operating at an experimental frequency of 23 MHz, was employed in conformity with the Petro-China Standard SY/T 6490–2007. The NMR instrument was fitted with a 30mm diameter probe, adept at minimizing the acquisition latency to a mere 15 μs, thereby facilitating the quantification of the minimal T_2_ response of the solid organic constituents in the shale. The CPMG (Carr-Purcell-Meiboom-Gill) sequence was employed to deduce the T_2_ spectrum. Concurrently, the two-dimensional T_1_-T_2_ relaxation correlation analyses were orchestrated leveraging the IR (Inversion Recovery)-CPMG sequence. The configuration for the CPMG sequence measurements comprised: waiting time TW = 2000 ms, echo time TE = 0.07 ms, number of scans NS = 16, and echo count NECH = 4000.

### 3.6 Fractal theory

Introduced by mathematician B. B. Mandelbrot in 1975, fractal theory offers a framework to elucidate intricate and irregular systems [7, 28]. The pronounced heterogeneity inherent to shale reservoirs poses challenges for conventional methodologies in accurately portraying their multifaceted pore architectures. Consequently, since the advent of fractal theory, numerous researchers have harnessed its principles to characterize shale’s pore structure, yielding commendable outcomes. As per the tenets of fractal theory, the fractal dimension of pores lies between 2 and 3. A higher fractal dimension indicates a more rugged pore surface and a more intricate pore configuration. In contrast, a diminished fractal dimension signifies a more rudimentary pore structure [29, 30].

Drawing from LTNA experiments, several models have been employed to compute fractal dimensions, encompassing the Langmuir model, BET model, and FHH model. Notably, the FHH model stands out as the most prevalently adopted model for fractal dimension calculations. The computational formula is delineated below:

$$\ln\;V=K\left[ln(ln(\frac{P}{P_{0}}))\right]+C$$

Where V is the volume of adsorbed N_2_ under pressure P, cm^3^/g; P and P_0_ are equilibrium pressure and saturation pressure, respectively, MPa; C is the intercept of the linear equation; K is the slope of the linear equation.

The value K can be derived from the plot of lnV versus $\left[ln(ln(\frac{P}{P_{0}}))\right]$;

Then fractal dimension D is given by D = K+3;

## 4 Results

### 4.1 Organic geochemistry characteristics

**Fig 2:**
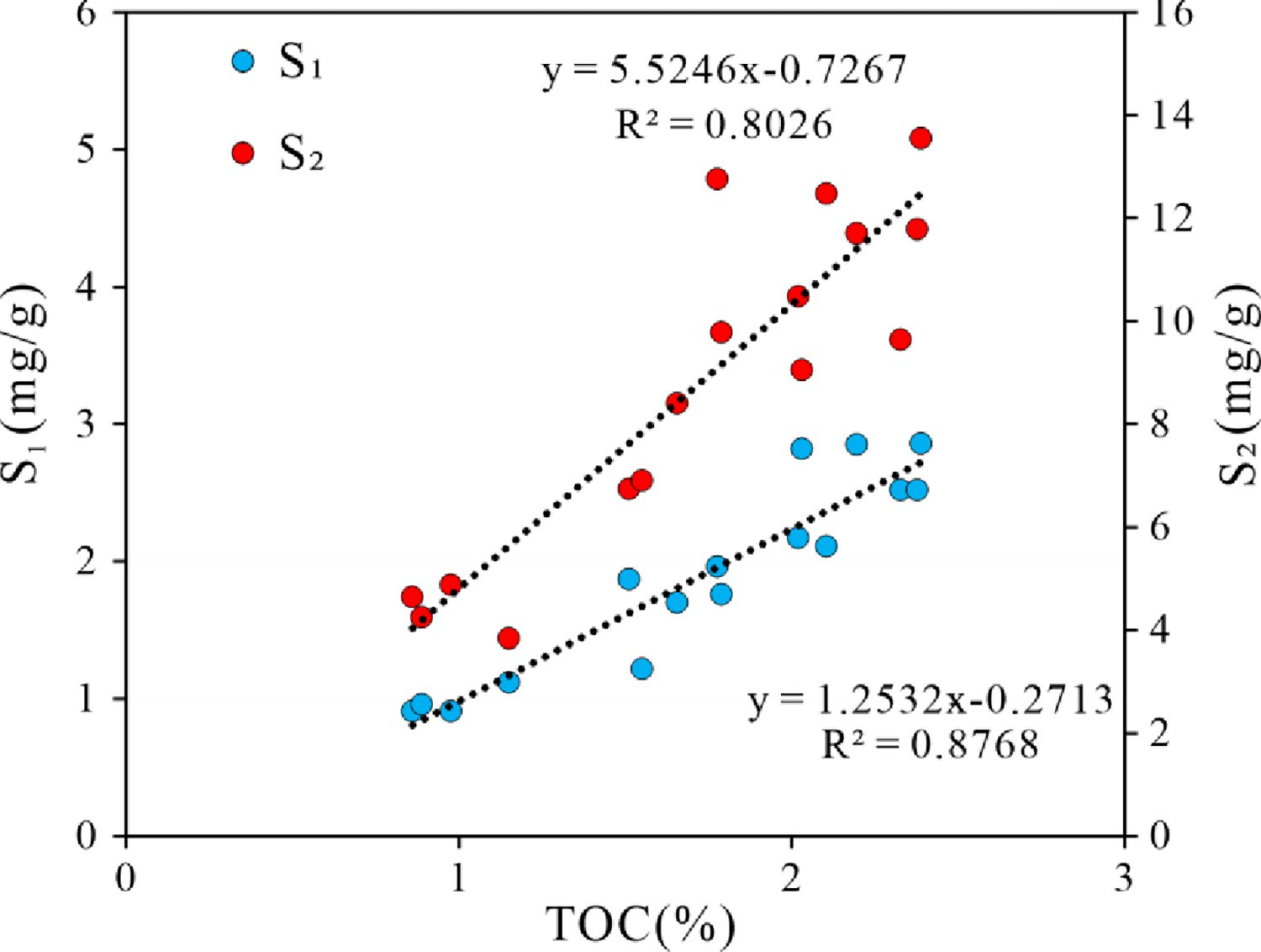
Plot of S_1_, S_2_ versus TOC. The T_max_ values fluctuate between 435°C and 454°C, with an average of 447°C. This suggests that the lacustrine shale samples have reached a mature phase in their thermal evolutionary trajectory. The hydrogen index, denoted as HI (HI = S_2_/TOC), spans from 334 mg/g to 718 mg/g, with a mean of 505 mg/g. The organic matter typology can be ascertained through a T_max_ versus HI plot [31]. As illustrated in Fig 3, the kerogen classifications for the Qingshankou Formation’s lacustrine shales predominantly align with Type I. This underscores the exceptional oil generation potential of the lacustrine shales in the study area.

Table 1 delineates the TOC and Rock-Eval pyrolysis parameters for the sixteen lacustrine shale specimens. The TOC values oscillate between 0.86% and 2.42%, averaging at 1.73%. The S_1_ content spans from 0.91 mg/g to 2.85 mg/g, with a mean value of 1.84 mg/g, while the S_2_ content exhibits a range from 3.84 mg/g to 13.55 mg/g, with a mean value of 8.81 mg/g. There is a significant positive correlation between S_1_, S_2_ and TOC, with the correlation coefficient R^2^ is 0.7993 and 0.8046 respectively (Fig 2).

**Fig 3:**
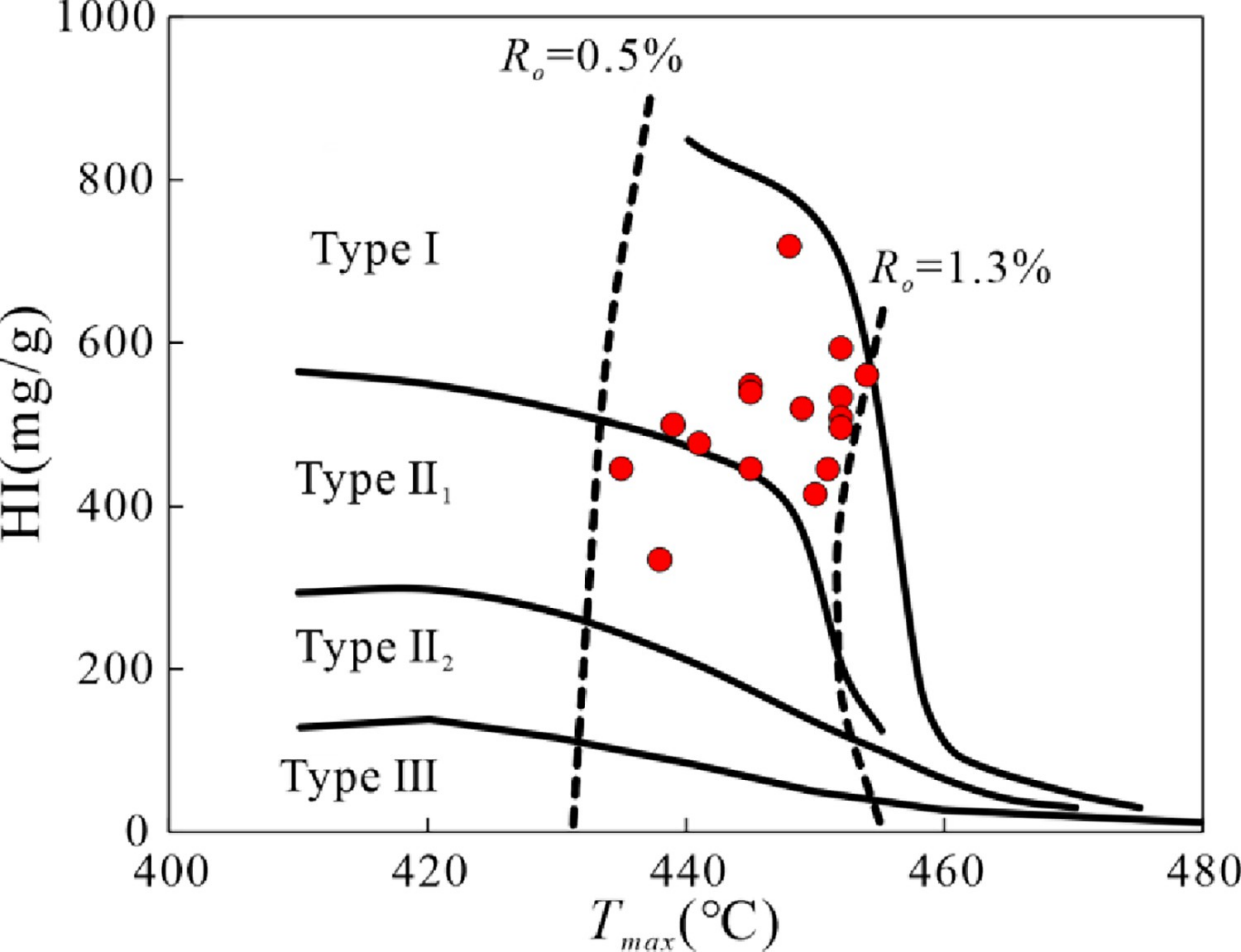
Plot of T_max_ versus HI.

**Table 1:** Organic geochemistry of the K_2_qn^1^ lacustrine shale samples.

Sample No.	Sample ID	Depth (m)	TOC (%)	T_max_ (°C)	S₁ (mg/g)	S₂ (mg/g)	S_1_+S_2_ (mg/g)	HI (mg/g)
**1**	D86-4	1971.2	1.78	448	1.96	12.76	14.72	718
**2**	D86-6	1974.2	1.79	445	1.76	9.79	11.55	547
**3**	D86-8	1976.9	0.86	445	0.91	4.64	5.55	539
**4**	D86-11	1979.6	0.98	439	0.94	4.87	5.81	499
**5**	D86-12	1982.2	0.89	441	0.96	4.24	5.20	477
**6**	D86-22	1984.3	1.15	438	1.12	3.84	4.96	334
**7**	D86-25	1985.7	2.02	449	2.14	10.49	12.63	519
**8**	D86-26	1987.6	1.51	445	1.87	6.74	8.61	446
**9**	D86-27	1989.7	2.33	450	2.42	9.64	12.06	414
**10**	D86-28	1990.8	2.03	435	2.82	9.06	11.88	446
**11**	D86-31	1993.9	2.20	452	2.85	11.71	14.56	533
**12**	D86-32	1996.3	1.66	452	1.70	8.41	10.11	508
**13**	D86-34	1999.5	2.11	452	2.11	12.48	14.59	593
**14**	D86-36	2001.8	2.38	452	2.52	11.79	14.31	496
**15**	D86-37	2003.7	2.42	454	2.06	13.55	15.61	560
**16**	D86-38	2007.0	1.55	451	1.22	6.90	8.12	445

Abbreviations: TOC = total organic carbon; T_max_ = Temperature at which maximum hydrocarbon generation; S_1_ = volatile hydrocarbon content; S_2_ = remaining hydrocarbon content; HI = hydrogen index (S_2_/TOC*100).

### 4.2 2D-NMR

**Fig 4:**
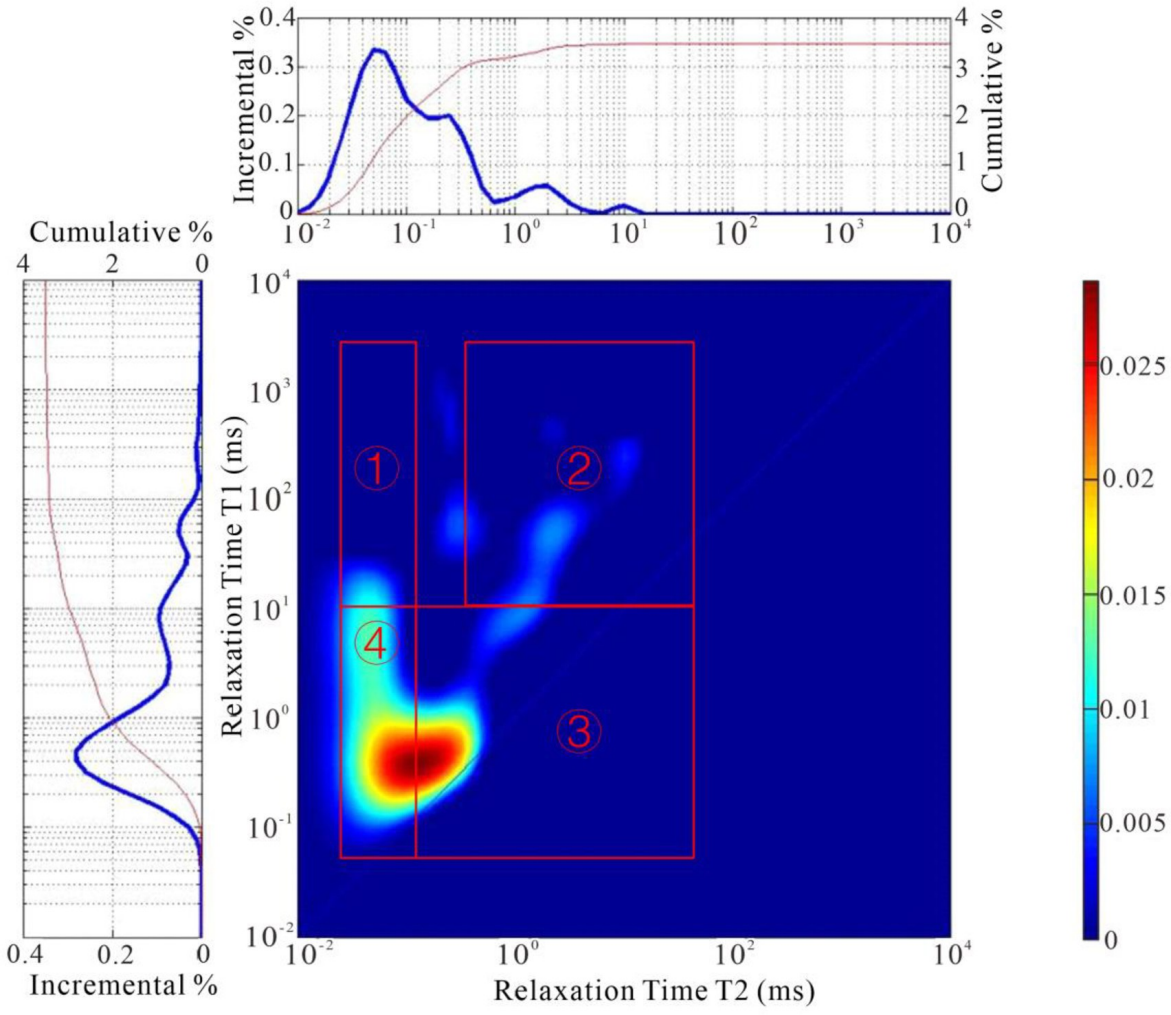
2D NMR spectrums showing 1H compounds in shale sample D86-31, 1993.9 m. Table 2 delineates the data: The content of solid organic matter ranged from 0.19–3.39 μl/g, averaging at 1.81 μl/g. The concentration of light oil spanned between 1.15–6.33 μl/g, with a mean value of 3.10 μl/g. The hydroxyl content varied from 13.96–28.92 μl/g, settling at an average of 21.93 μl/g, while the water content within pore systems fluctuated from 2.10–13.39 μl/g, with an average content of 7.21 μl/g.

Taking sample D86-31 as a representative example, Fig 4 presents the T_1_-T_2_ map, elucidating the distribution of various 1H phases within the shale. Based on prior studies [32, 33], the T_1_-T_2_ map is demarcated into four distinct signal intervals. Zone 1, positioned at the top-left quadrant of Fig 4, signifies the presence of solid organic matter, encompassing kerogen, solid asphalt, heavy oil, and the like. Conversely, Zone 2, located in the top-right quadrant of Fig 4, denotes the signal region for soluble organic matter and is conventionally associated with light oil. Zone 3, situated at the bottom-left of Fig 4, pertains to hydroxyl-rich compounds, while Zone 4, in the bottom-right quadrant of Fig 4, corresponds to the water signal evident within the pore systems.

**Table 2:** Fluid contents of different phases derived from 2D-NMR measurement.

Sample No.	Sample ID	Depth (m)	Solid OM (μl/g)	Hydroxyls (μl/g)	Water (μl/g)	Light oil (μl/g)
**1**	D86-4	1971.2	1.31	20.62	3.54	3.30
**2**	D86-6	1974.2	2.55	22.78	10.92	2.72
**3**	D86-8	1976.9	0.19	20.28	5.30	1.15
**4**	D86-11	1979.6	0.55	18.33	2.78	1.58
**5**	D86-12	1982.2	0.32	13.96	3.21	1.31
**6**	D86-22	1984.3	0.62	17.39	5.69	1.60
**7**	D86-25	1985.7	2.18	17.52	2.10	3.61
**8**	D86-26	1987.6	0.91	27.76	4.40	2.69
**9**	D86-27	1989.7	2.91	20.12	7.07	3.75
**10**	D86-28	1990.8	2.58	27.66	10.94	5.56
**11**	D86-31	1993.9	3.32	27.02	13.39	6.33
**12**	D86-32	1996.3	1.03	28.92	7.13	2.33
**13**	D86-34	1999.5	2.21	26.00	9.44	3.97
**14**	D86-36	2001.8	3.13	22.40	10.34	4.59
**15**	D86-37	2003.7	3.39	18.15	6.30	2.83
**16**	D86-38	2007.0	1.77	21.92	12.82	2.33

Abbreviations: OM = Organic Matter

**Fig 5:**
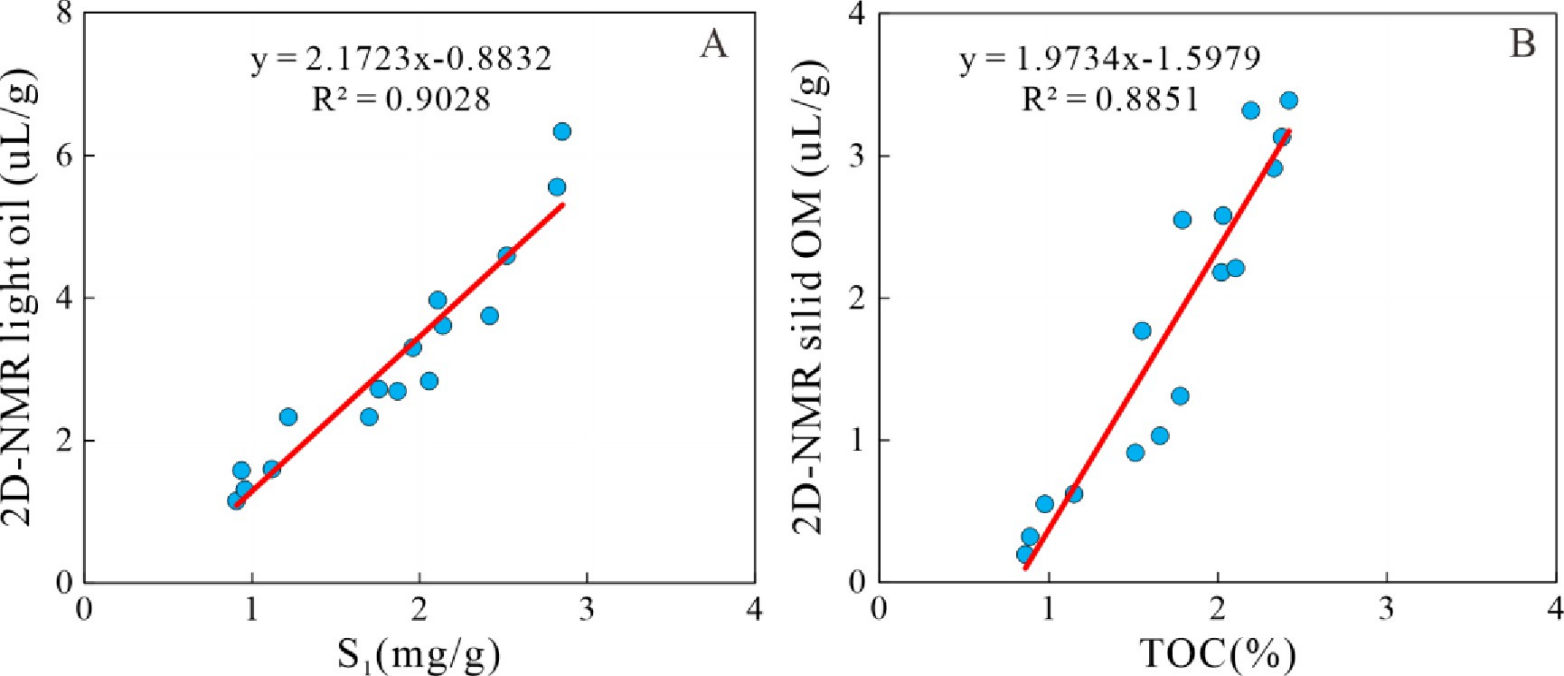
Relationships between geochemistry parameters and 2D-NMR parameters. A: Plot of 2D-NMR light oil content and S_1_; B: Plot of 2D-NMR solid OM content and TOC.

A robust linear correlation was discerned between the 2D-NMR light oil content and S_1_, as evidenced in Fig 5A (R^2^ = 0.9028). This corroborates the precision of the 2D NMR technique in characterizing shale oil content. Similarly, a compelling linear association was observed between the content of solid organic matter and TOC, as depicted in Fig 5B (R^2^ = 0.8851), suggesting the viability of employing 2D NMR measurements to assess organic matter content.

### 4.3 Mineralogy

**Fig 6:**
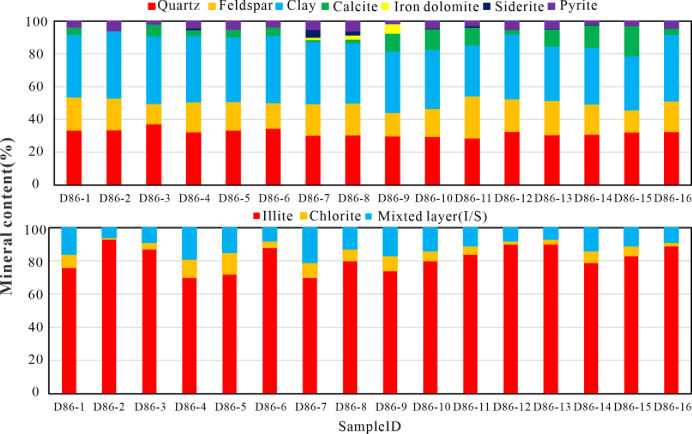
Mineral compositions of the K_2_qn^1^ lacustrine shale samples.

As delineated in Table 3 and illustrated in Fig 6, XRD analyses reveal that the predominant mineralogical constituents of the lacustrine shale specimens from the study area encompass clay minerals, quartz, and feldspar. Clay minerals emerge as the most abundant, with concentrations fluctuating between 31.2% and 41.3%, and an average value of 37.6%. Quartz content varies from 28.5% to 37.3%, averaging at 31.7%. Feldspar content spans from 11.3% to 24.8%, with a median value of 18.5%. The concentrations of other minerals remain relatively subdued, not exceeding 10%.

**Table 3:** Mineralogical compositions of the K_2_qn^1^ lacustrine shale samples.

No.	Sample ID	Depth (m)	Quartz (%)	Feldspar (%)	Calcite (%)	Iron dolomite (%)	Siderite (%)	Pyrite (%)	Clay (%)	Illite (%)	Chlorite (%)	Mixed layer (I/S)	Lithology
**1**	D86-4	1971.18	33.5	20.1	4.5	/	/	3.7	38.2	76	8	16	S-3
**2**	D86-6	1974.22	37.3	15.7	0.4	/	/	5.8	40.8	93	1	6	S-3
**3**	D86-8	1976.86	32.2	17.5	7.3	/	/	1.9	41.1	87	4	9	M-2
**4**	D86-11	1979.55	30.9	19.7	3.7	/	/	4.3	40.4	70	11	19	S-3
**5**	D86-12	1982.21	28.6	22.2	4.7	/	/	5.0	39.5	72	13	15	S-3
**6**	D86-22	1984.25	30.6	19.5	5.0	/	/	3.6	41.3	88	4	8	S-3
**7**	D86-25	1985.73	30.6	19.0	1.2	1.5	4.7	5.2	37.8	70	9	21	M-2
**8**	D86-26	1987.62	28.5	21.4	2.4	2.5	2.3	6.2	36.7	80	7	13	M-2
**9**	D86-27	1989.67	28.8	15.4	10.9	5.7	/	1.7	37.5	74	9	17	M-2
**10**	D86-28	1990.81	33.7	13.0	12.6	/	0.5	4.3	35.9	80	6	14	M-2
**11**	D86-31	1993.93	29.5	24.8	10.6	/	1.1	2.8	31.2	84	5	11	S-3
**12**	D86-32	1996.3	33.5	19.1	2.6	/	/	5.3	39.5	90	2	8	S-3
**13**	D86-34	1999.46	32.3	19.3	10.4	/	0.3	4.6	33.1	90	3	7	S-3
**14**	D86-36	2001.83	30.7	18.7	13.7	/	0.4	2.1	34.4	79	7	14	M-2
**15**	D86-37	2003.73	34.5	11.3	18.2	/	/	2.9	33.1	83	6	11	M-2
**16**	D86-38	2006.98	32.7	18.5	3.7	/	/	4.5	40.6	89	2	9	S-3

Within the clay mineral category, illite dominates, with proportions ranging from 70% to 93% and an average of 81.6%. This is succeeded by the mixed layer (I/S), which oscillates between 6% and 21%, with a mean of 12.4%. Additionally, trace amounts of chlorite are present, ranging from 1% to 13%, with an average concentration of 6.1%.

**Fig 7:**
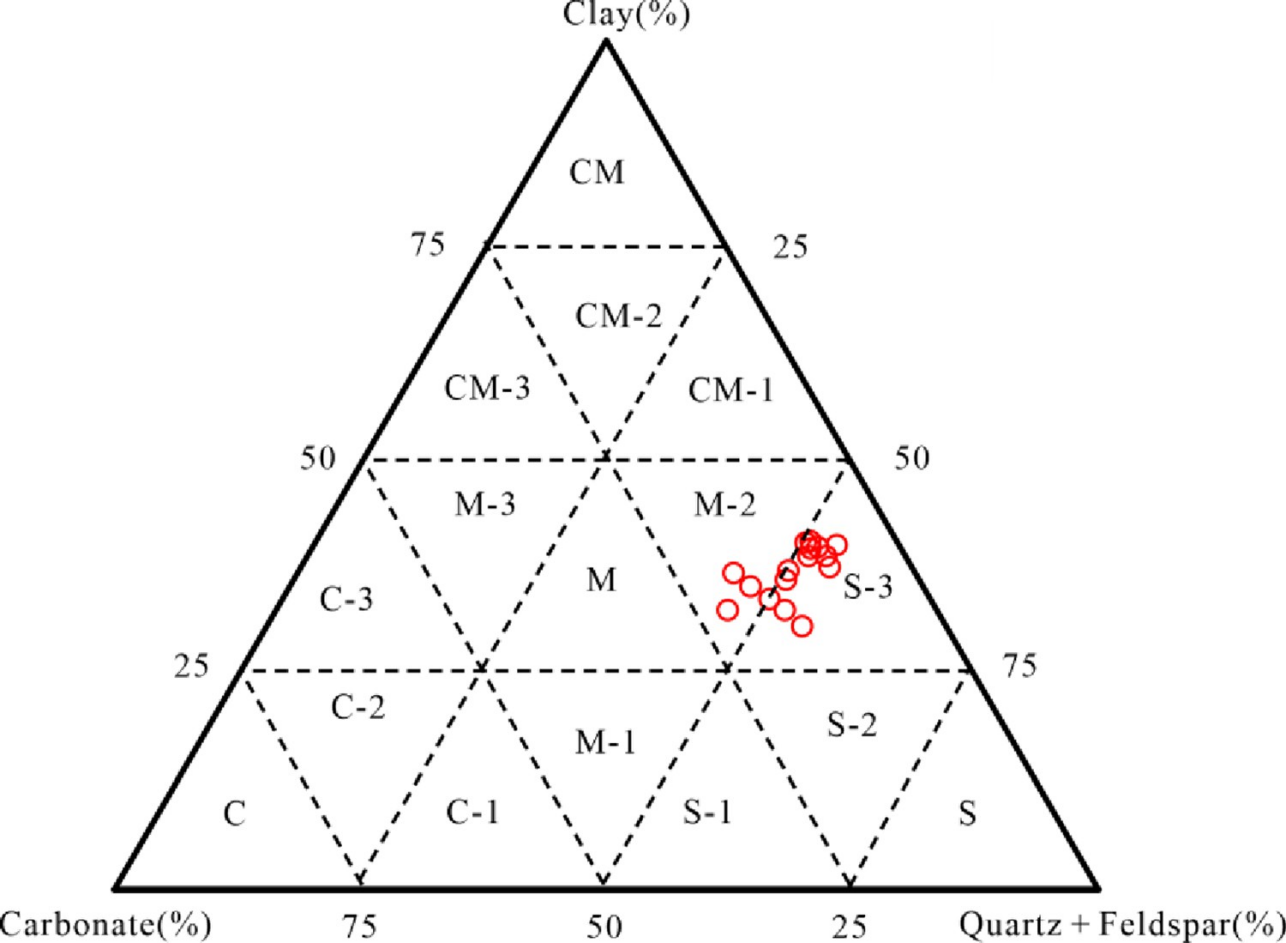
Classification of shale lithofacies of the K_2_qn^1^ lacustrine shale samples.

Employing a ternary plot encompassing siliceous minerals, carbonate minerals, and clay minerals, sixteen lithofacies have been demarcated [15, 34]. Fig 7 presents the lithofacies categorization of the K_2_qn^1^ lacustrine shale specimens in the study area. Two distinct lithofacies have been discerned: the argillaceous/siliceous mixed shale lithofacies (M-2) and the argillaceous-rich siliceous shale lithofacies (S-3).

### 4.4 LTNA experiment

**Fig 8:**
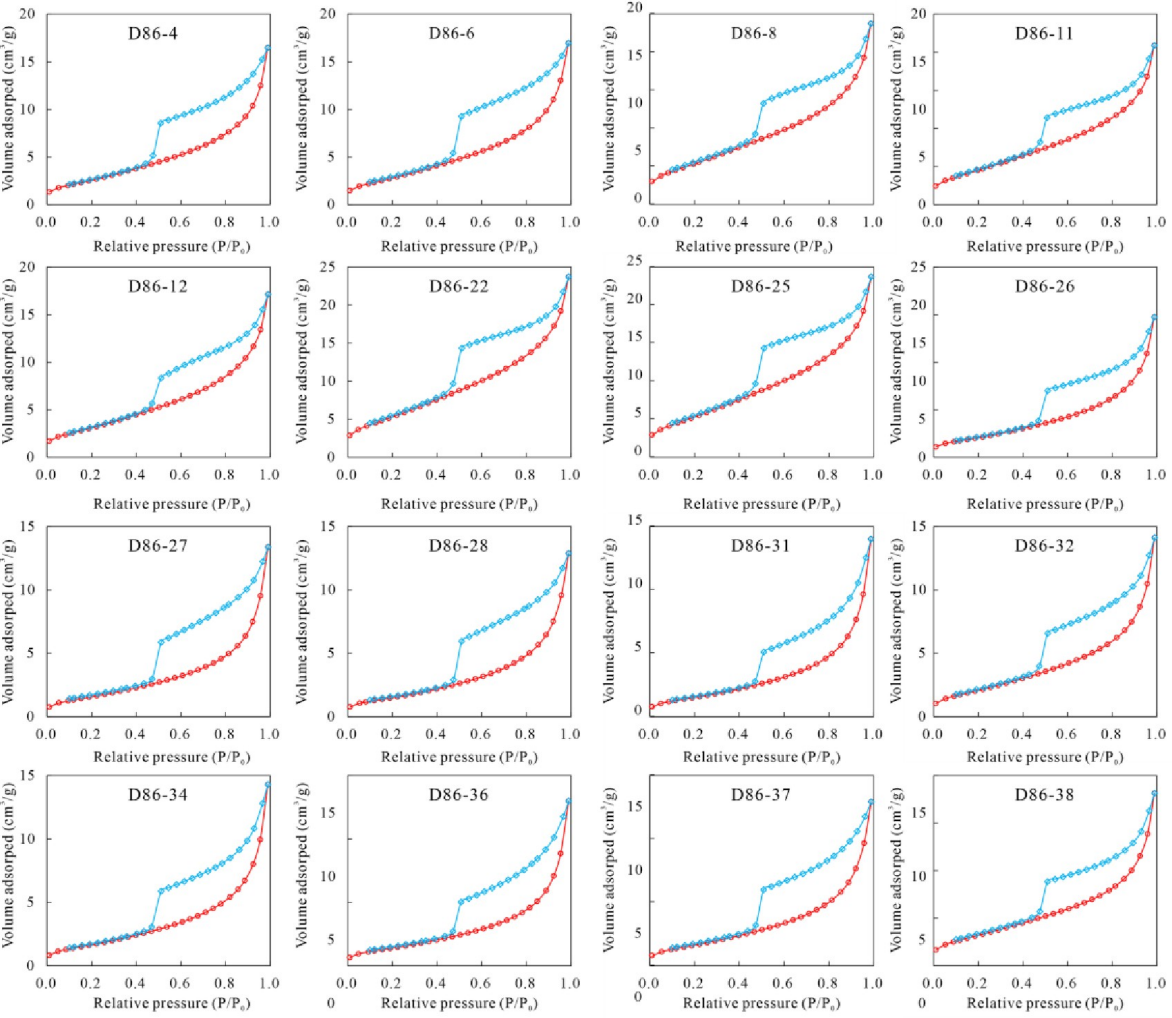
N_2_ adsorption and desorption isotherms of the K_2_qn^1^ lacustrine shale samples. The BET model can be applied to the SSA and average pore diameter (APD) of multi-scale pore media, while the premise assumption of the Kelvin’s equation is based on a single pore size. Therefore, for shale, a porous medium with a wide range of pore sizes, the effective model for obtaining SSA and APD should use the BET model, which is also widely used in the industry. For computational purposes, the BET model was utilized for the estimation of SSA and APD (BET-SSA and BET-APD), and the BJH model was employed to determine PV. As presented in Table 4, BJH-PV values oscillate between 0.0201 cm^3^/g and 0.0378 cm^3^/g, with a mean of 0.0256 cm^3^/g. BET-SSA values span from 5.47 m^2^/g to 20.60 m^2^/g, averaging at 10.17 m^2^/g. Concurrently, BET-APD values range from 5.60 nm to 9.79 nm, averaging at 7.81 nm.

The LTNA method is a universally acknowledged methodology for elucidating the architecture of mesopores, typically ranging from 2 nm to 50 nm [31]. As depicted in Fig 8, the LTNA adsorption and desorption isotherms manifest an inverse "S" configuration, aligning with the archetypal IUPAC Type IV isotherm. The adsorption and desorption isotherms separate at P/P_0_ = 0.5, forming a significant hysteresis loop, attributable to capillary condensation within mesopore structures. In accordance with IUPAC’s classification, the hysteresis loop of the sixteen K_2_qn^1^ lacustrine shale samples have the characteristics of preposition of H2 and H3, indicating the presence of inkbottle-shaped pore with narrow necks and slit-shaped pores [35].

**Table 4:** LTNA pore structure parameters and fractal dimensions of the K_2_qn^1^ lacustrine shale samples.

Sample No.	Sample ID	Depth (m)	BJH-PV	BET-SSA	BET-APD	D_1_	D_2_
**1**	D86-4	1971.2	0.0257	10.03	7.36	2.6695	2.3451
**2**	D86-6	1974.2	0.0265	10.76	7.25	2.6824	2.3606
**3**	D86-8	1976.9	0.0378	20.23	5.64	2.7460	2.4209
**4**	D86-11	1979.6	0.0332	16.99	5.88	2.7401	2.3987
**5**	D86-12	1982.2	0.0266	12.00	6.60	2.7083	2.3924
**6**	D86-22	1984.3	0.0377	20.60	5.60	2.7551	2.3937
**7**	D86-25	1985.7	0.0237	8.35	8.19	2.6441	2.3454
**8**	D86-26	1987.6	0.0230	7.88	8.20	2.6414	2.3713
**9**	D86-27	1989.7	0.0208	6.05	9.17	2.6029	2.3383
**10**	D86-28	1990.8	0.0201	5.66	9.79	2.5976	2.3618
**11**	D86-31	1993.9	0.0218	5.80	9.58	2.5715	2.3520
**12**	D86-32	1996.3	0.0220	8.00	7.63	2.6596	2.3429
**13**	D86-34	1999.5	0.0223	6.38	9.10	2.5980	2.3545
**14**	D86-36	2001.8	0.0202	5.47	9.56	2.5827	2.3247
**15**	D86-37	2003.7	0.0202	6.33	8.81	2.6169	2.3441
**16**	D86-38	2007.0	0.0286	12.20	6.55	2.6866	2.3984

Abbreviations: BJH-PV = Pore Volume based on BJH model; BET-SSA = Specific Surface Area based on BET model; BET-APD = Average Pore Diameter calculated based on BET model. D = fractal dimension, D_1_ (0 < P/P_0_ < 0.5), D_2_ (0.5 <P/P_0_ <1.0).

### 4.5 Fractal dimensions from LTNA

**Fig 9:**
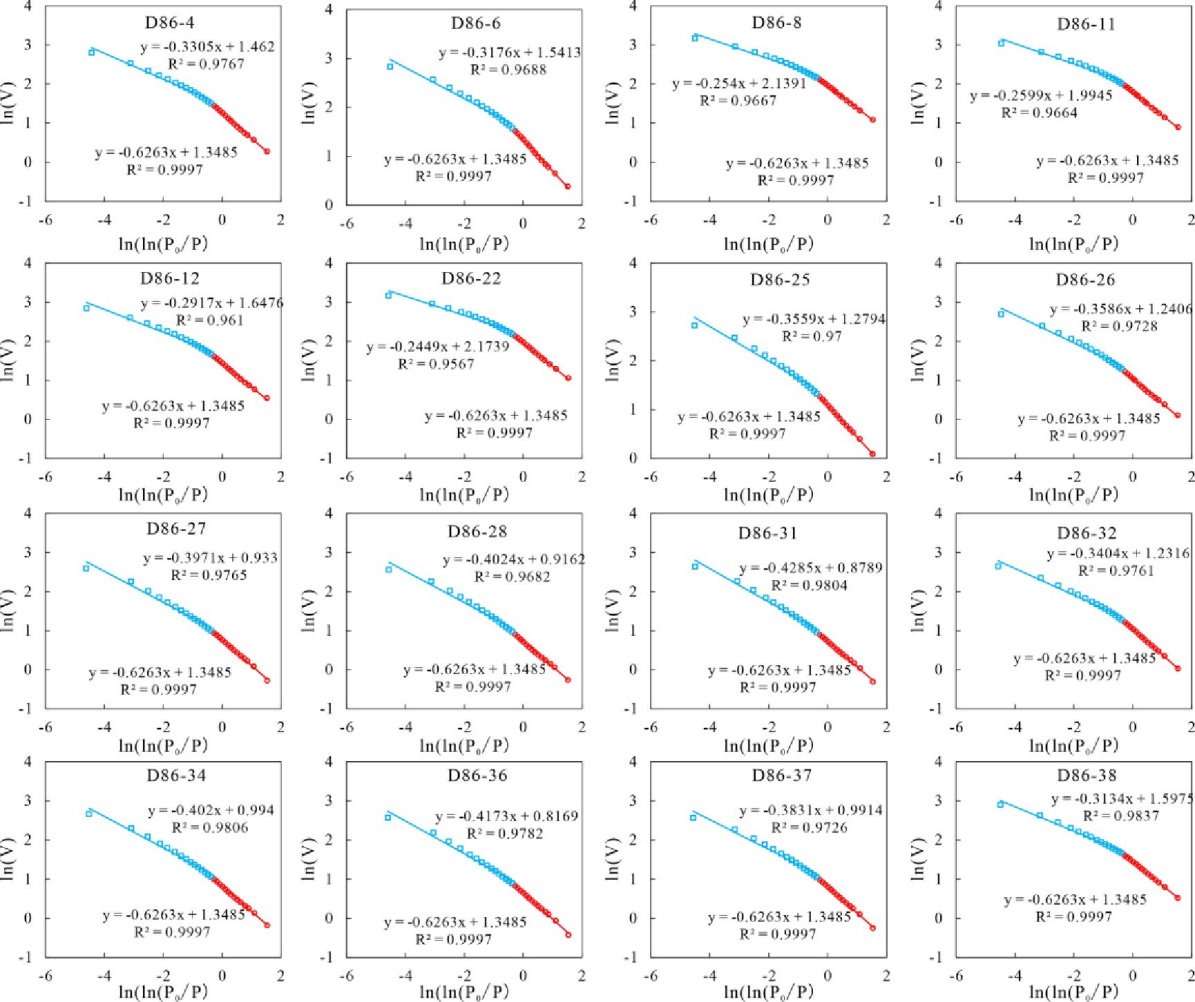
Fractal dimension calculation results with ln(V) versus ln(ln(P/P_0_)) from N_2_ adsorption isotherms. D_1_ fluctuates between 2.5715 and 2.7551, with the average of 2.6564; while D_2_ ranged from 2.3247 to 2.4209, with the average of 2.3653. Notably, D_1_ consistently surpasses D_2_, suggesting that smaller pores exhibit greater homogeneity compared to their larger counterparts (refer to Table 4).

The plots of lnV versus $\left[ln(ln(\frac{P}{P_{0}}))\right]$ of all sixteen K_2_qn^1^ lacustrine shale samples are depicted in Fig 9. Taking P/P_0_ = 0.5 as the boundary, two distinct fractal dimensions emerge within the pore systems, which is during to the different adsorption mechanisms of the two types of pores. Therefore, two fractal dimensions D_1_ (0 < P/P_0_ < 0.5) and D_2_ (0.5 <P/P_0_ <1.0) were calculated.

## 5. Discussion

### 5.1 Relationships between fractal dimensions and pore structure parameters

**Fig 10:**
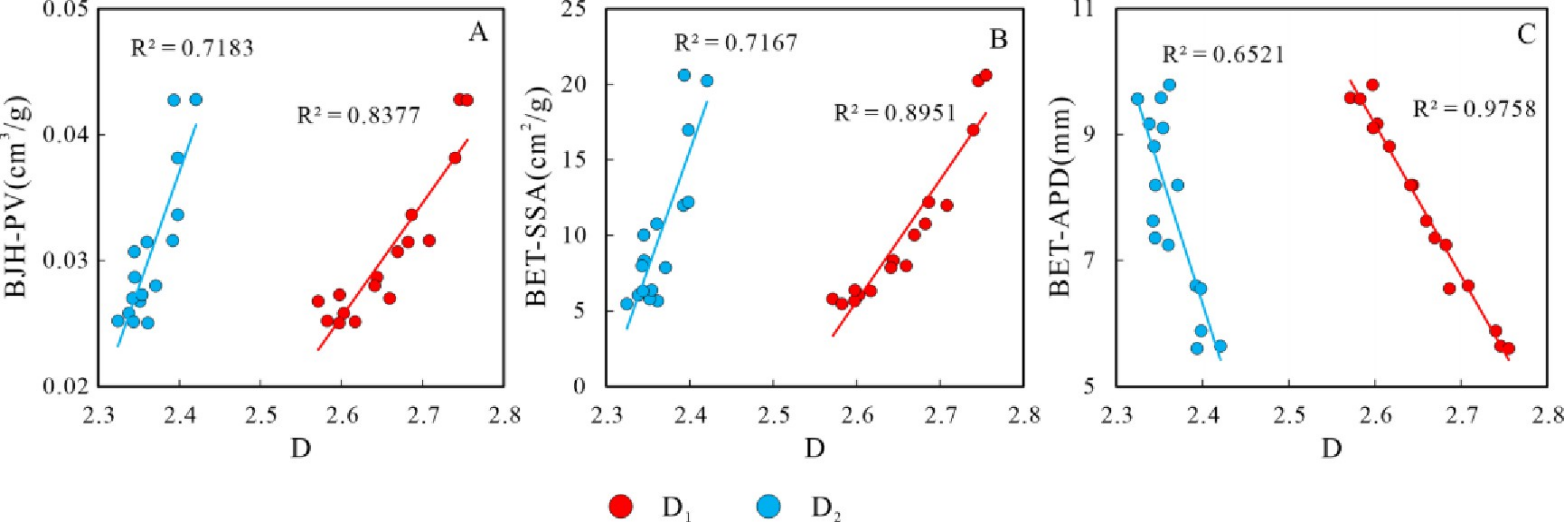
Relationships between pore structure parameters and fractal dimensions. Notably, the correlation coefficient R^2^ of D_1_ with BJH-PV, BET-SSA, and BET-APD is markedly superior to that of D_2_. This underscores the aptness of D_1_ in characterizing the microporous architecture of the K_2_qn^1^ lacustrine shale specimens from the designated study region.

As delineated in Fig 10, the interrelationships between fractal dimensions and pore structural parameters were explored, namely BJH-PV, BET-SSA and BET-APD. Both fractal dimensions, D_1_ and D_2_, exhibit a positive correlation with BJH-PV and BET-SSA. This suggests that lacustrine shale specimens with augmented fractal dimensions consistently possess larger BJH-PV and BET-SSA values. Conversely, the BET-APD of the lacustrine shale specimens manifests an inverse relationship with fractal dimensions, with a particularly strong correlation for D_1_ (R^2^ = 0.9758). This observation aligns with experimental results from coal-bearing Cretaceous Nenjiang shales, marine shale in the Sichuan Basin, and lacustrine shale from the Bohai Bay Basin [36–38]. This is because the smaller the average pore diameters, the greater the specific surface area and pore volume, which leads to a more intricate pore structure, enhanced heterogeneity of shale reservoirs, and increased fractal dimensions [39–41].

### 5.2 Relationships between fractal dimensions and mineral composition, geochemistry parameters and 2D-NMR parameters

**Fig 11:**
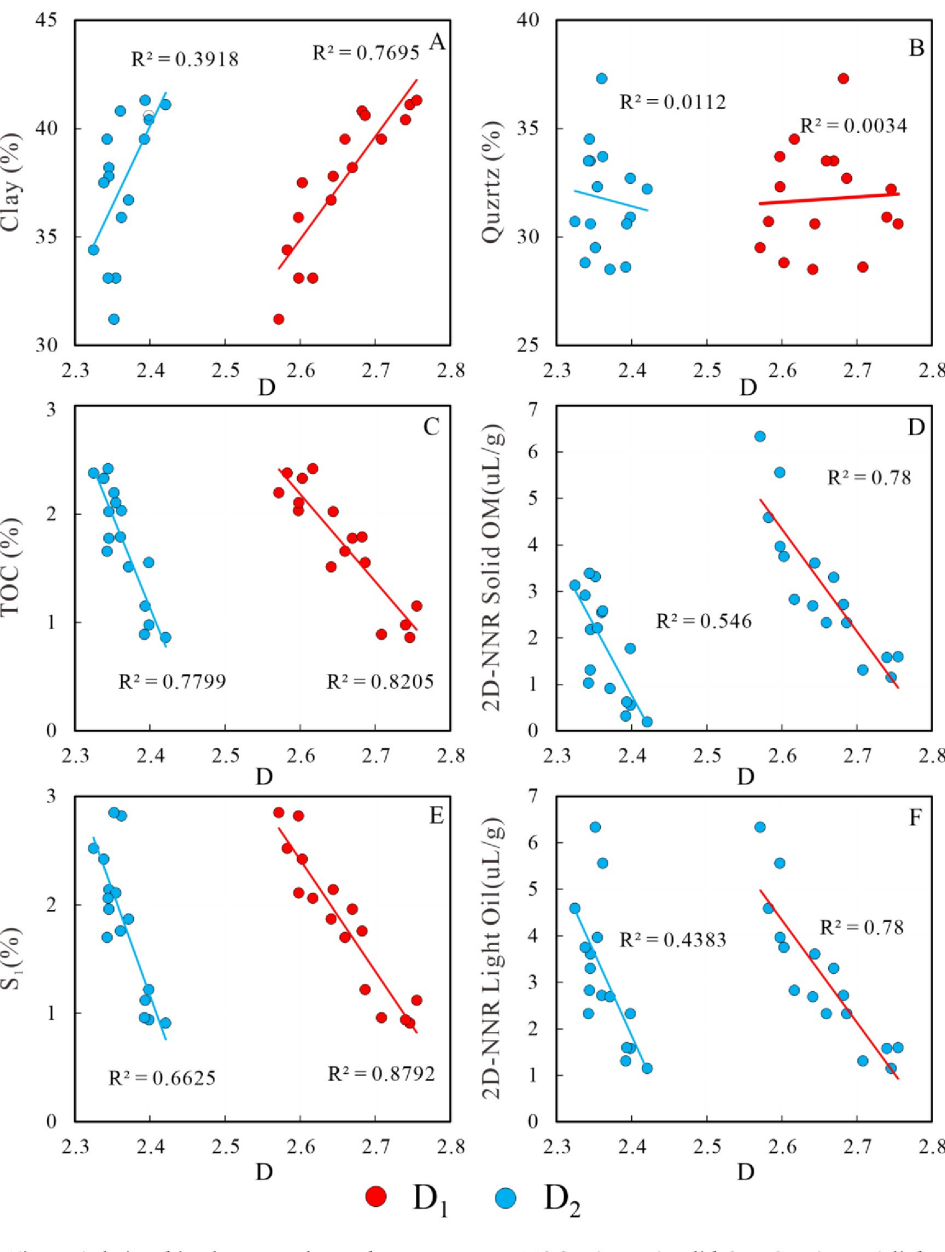
Relationships between clay and quartz content, TOC, 2D-NMR solid OM, S_1_, 2D-NMR light oil and fractal dimensions. Understanding the relationship between TOC and fractal dimension D holds significant importance in gaining a deeper understanding of the pore structure of shale reservoirs and the mechanism of shale oil enrichment. The correlation between fractal dimension D and TOC is depicted as negative in Fig 11C. As the TOC content escalates, the fractal dimensions diminish. Both D_1_ and D_2_ exhibit a robust inverse relationship with TOC, boasting correlation coefficients (R^2^) of 0.8205 and 0.7799, respectively. This phenomenon is similar to the data of lacustrine shale from Qingshankou Formation documented by Wang and Lacustrine Oil-Bearing Shale from the Dongying Sag documented by Zhang when the TOC content is less than 3%, but contrasts that of the aforementioned gas marine shale [7, 37, 42]. The reasons for this phenomenon can be attributed to several factors. In shale within the oil window, the organic matter contains relatively few pores, which means that the pore structure and surface remain largely unaffected by the organic matter itself. Consequently, the fractal dimensions do not increase with rising total organic carbon (TOC) content. Furthermore, as the organic matter content (TOC) increases, a greater quantity of organic acids is generated during the hydrocarbon generation process. This results in the formation of larger dissolution pores within the carbonated minerals [43], leading to a decrease in the fractal dimensions D_1_ and D_2_. There is also a negative correlation between fractal dimension D and 2D-NMR OM content (Fig 11D). This suggests that both fractal dimensions are apt for characterizing the TOC content.

Quartz and clay are the predominant mineral types in the lacustrine shale oil reservoir of the Qingshank ou Formation in the study area, constituting an average total proportion of 69.3% (Fig 6 and Table 3). As depicted in Fig 11A, there is a positive correlation between the content of clay and fractal dimension D, particularly for D_1_ (R^2^ = 0.7695). With an increase in clay content, the fractal dimension D gradually increases. This complexity arises due to the filling of intergranular pores by clay minerals, resulting in increased reservoir heterogeneity. Therefore, fractal dimension can serve as a characterization parameter for reservoir heterogeneity, where a smaller fractal dimension indicates stronger reservoir homogeneity. There is no clear correlation between quartz and fractal dimension D, suggesting that quartz has minimal impact on the pore structure and heterogeneity of the lacustrine shale reservoirs (Fig 11B). This finding aligns with the conclusions drawn from Wang Min’s research results.

The S_1_ content is a critical parameter in shale oil evaluation, and it indicates shale oil potential [44–51]. Analogous to the correlation between fractal dimensions and TOC/2D-NMR OM content, S_1_ content/2D-NMR light oil content diminishes with an uptick in fractal dimensions (Fig 11E and 11F). This trend is particularly pronounced for D_1_. Conversely, the inverse relationship between D_2_ and S_1_ is relatively tenuous, with an R^2^ value of 0.6625. This implies that oil predominantly occupies the smaller pores, establishing D_1_ as an exemplary parameter for delineating the S_1_ content and 2D-NMR light oil content.

## 6. Conclusions

1. The lacustrine shale samples from the Upper Cretaceous Qingshankou Formation in the Southern Songliao Basin exhibit significant organic richness. The mean values for TOC, S_1_, S_2_, and S_1_+S_2_ stand at 1.73%, 1.84mg/g, 8.81mg/g, and 10.64mg/g, respectively.
2. The primary mineralogical constituents are clay, quartz, and feldspar, with respective average contents of 37.6%, 31.7%, and 18.5%. The analysis discerned two lithofacies: the argillaceous/siliceous mixed shale lithofacies (M-2) and the argillaceous-rich siliceous shale lithofacies (S-3).
3. Two fractal dimensions D_1_ (0 < P/P_0_ < 0.5) and D_2_ (0.5 <P/P_0_ <1.0) were obtained from LTNA isotherms using FHH method. D_1_ ranges from 2.5715 and 2.7551 (averaged 2.6564), D_2_ spans from 2.3247 to 2.4209 (averaged 2.3653).
4. The fractal dimension D_1_ exhibits a progressive increase with the augmentation of clay content, suggesting a significant impact of clay on the heterogeneity of shale oil reservoirs. The presence of clay minerals undermines the efficacy of hydraulic fracturing due to their propensity for water absorption and subsequent expansion, thereby adversely affecting the enrichment, migration, and development of shale oil. Consequently, an escalation in clay content is associated with detrimental effects on shale oil potential.
5. Both D_1_ and D_2_ demonstrate a positive correlation with BJH-PV and BET-SSA, while a they escalated with diminishing BET-APD. Additionally, a decline in both dimensions is observed with an increase in TOC, 2D-NMR solid organic matter (OM), S_1_ content and 2D-NMR light oil. This trend highlights the influence of reservoir heterogeneity on shale oil content, particularly the content of movable oil, which tends to increase as reservoir heterogeneity decreases. Therefore, in the context of shale oil development, the fractal dimensions D, can be instrumental in evaluating shale oil targets. Among them, D_1_, in particular, proves to be a more precise parameter for elucidating pore structure complexities and assessing the shale oil potential of the K_2_qn^1^ lacustrine shale samples within the specified study area.

## Supporting information

S1 Data (XLSX)
